# Transvenous Embolization for a Diploic Arteriovenous Fistula Presenting With Tinnitus: A Case Report

**DOI:** 10.7759/cureus.93261

**Published:** 2025-09-26

**Authors:** Kazuhide Maeshima, Hideki Takemoto, Hirotaka Yamanaka, Nobuyuki Miyatake

**Affiliations:** 1 Neurological Surgery, Japanese Red Cross Wakayama Medical Center, Wakayama, JPN

**Keywords:** arteriovenous fistula [avf], bilateral pulsatile tinnitus, endovascular coil embolization, intraosseous arteriovenous fistula, transvenous embolisation

## Abstract

Intraosseous arteriovenous fistula (AVF) is rare, and diploic AVF is an even rarer subtype with very few reported cases. We describe a 30-year-old man who presented with bilateral pulsatile tinnitus. Magnetic resonance imaging (MRI) revealed abnormal signals in the occipital region, prompting digital subtraction angiography (DSA), which demonstrated arteriovenous shunting from transosseous branches of both occipital arteries (OAs) converging at a single point within the diploë. The shunted flow drained into the transverse sinus via a diploic vein. Although venous drainage was antegrade without cortical reflux, the patient experienced intolerable tinnitus. Transvenous embolization (TVE) was performed targeting the outflow vein immediately distal to the shunt point, where all shunted flow converged. This resulted in rapid resolution of tinnitus without neurological deficits. This case highlights an exceedingly rare instance of occipital diploic AVF in the posterior fossa and emphasizes that early recognition and definitive endovascular embolization can achieve a curative outcome before complications develop.

## Introduction

Intraosseous arteriovenous fistula (AVF) is a rare type of intracranial arteriovenous shunt disorder. Among these, intraosseous AVFs involving the diploic veins, referred to as diploic AVFs, are even more uncommon, with fewer reported cases in the literature than typical intraosseous AVFs. While most reported cases involve the diploic veins of the frontal region [1-5], occurrences in the occipital bone are exceedingly rare.

Clinically, patients with diploic AVFs may present with symptoms, such as pulsatile tinnitus, headache, or cortical manifestations, depending on the venous drainage pattern.

We report the case of a 30-year-old Japanese man who presented with tinnitus and was diagnosed with a diploic AVF in the occipital bone. Transvenous embolization (TVE) was performed, leading to rapid symptom resolution.

## Case presentation

**Figure 1:**
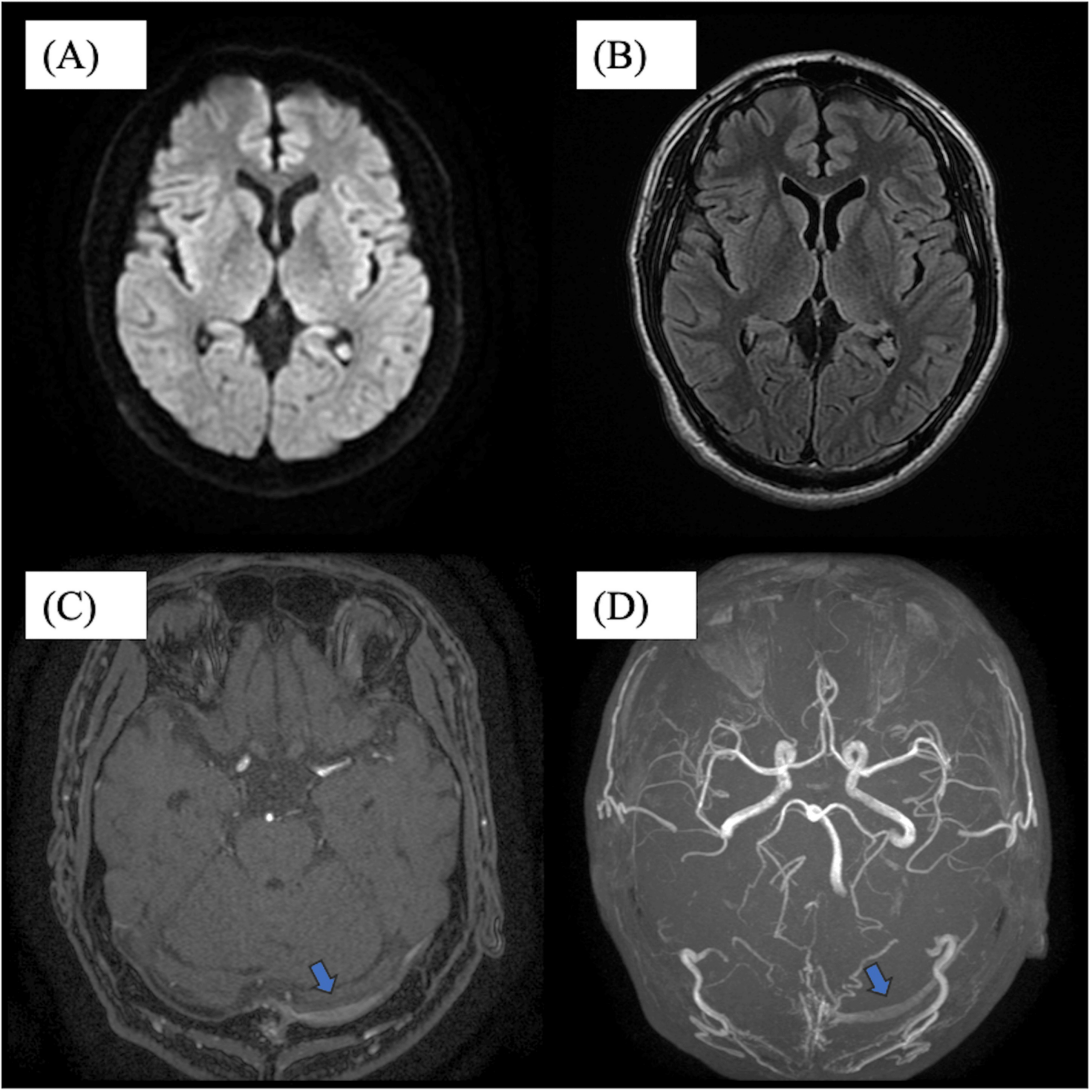
MRI and magnetic resonance angiography (MRA) showing abnormal vascular signals in the left transverse sinus. (A) MRI diffusion-weighted imaging (DWI) showed no abnormal findings. (B) MRI fluid-attenuated inversion recovery (FLAIR) showed no abnormal findings. (C) Time-of-flight (TOF) MRA axial scan showing abnormal flow-related signal in the left transverse sinus (arrows). (D) TOF MRA 3D reconstruction showing abnormal flow-related signal in the left transverse sinus (arrows).

A 30-year-old Japanese man presented with bilateral pulsatile tinnitus, which he had first noticed in September of the previous year. Although he did not experience associated symptoms, such as headache, dizziness, or vomiting, the tinnitus gradually worsened and became intolerable. He therefore visited a local clinic in December. Magnetic resonance imaging (MRI) and magnetic resonance angiography (MRA) revealed abnormal signal intensities in the left transverse sinus, prompting referral to our institution (Figure 1).

**Figure 2:**
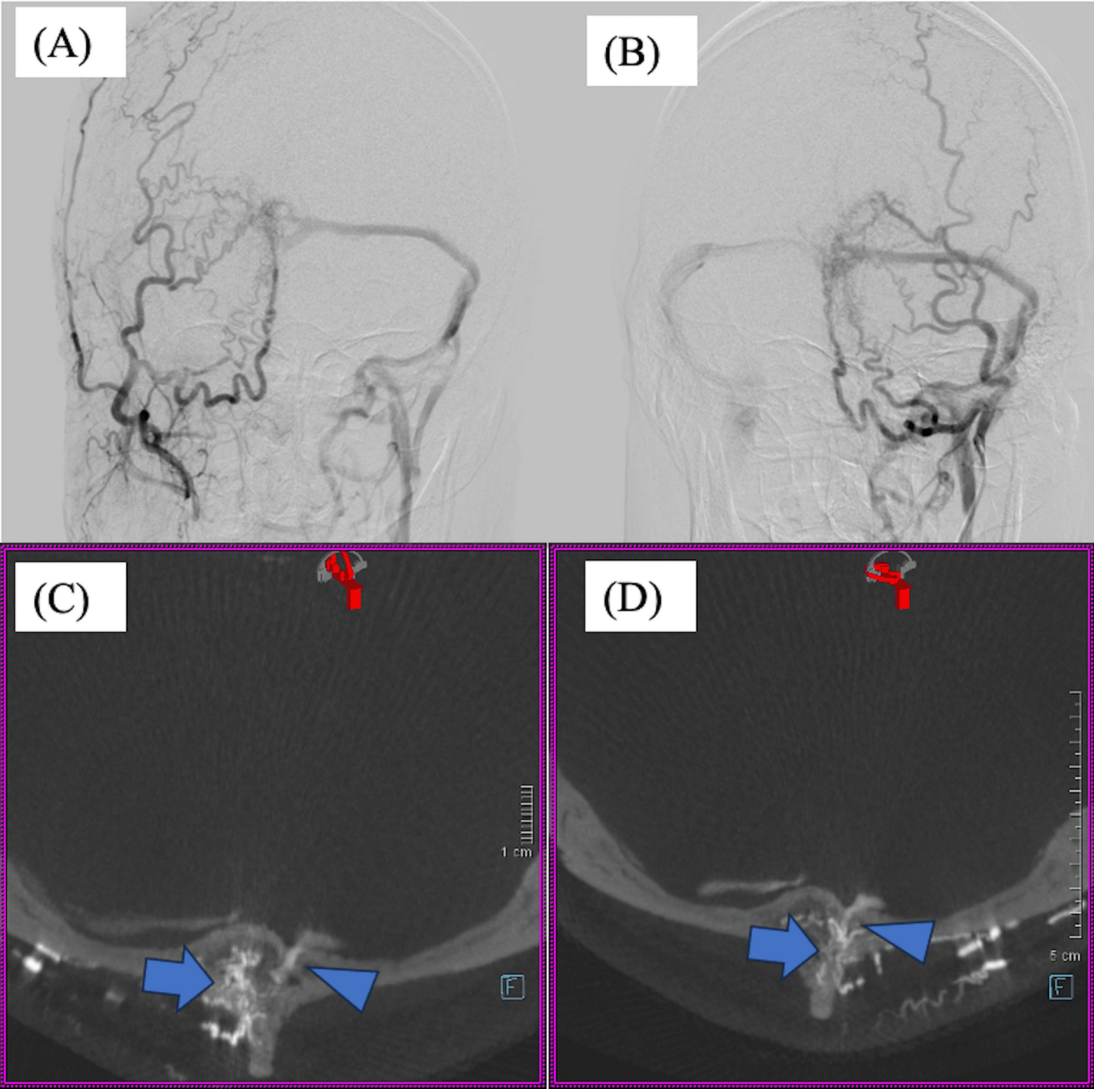
Digital subtraction angiography (DSA) demonstrating occipital diploic arteriovenous fistula (AVF) with drainage into the transverse sinus. (A) DSA of the right external carotid artery (ECA) injection showing transosseous branches of the right occipital artery (OA) converging at a single site to form a shunt draining into the transverse sinus via a diploic vein. (B) DSA of the left OA injection showing transosseous branches of the left OA converging at a single site to form a shunt draining into the transverse sinus via a diploic vein. (C) Maximum intensity projection (MIP) image from 3D rotational digital subtraction angiography (3D-DSA) of the right ECA shows the shunt point located within the diploic layer (arrow) and draining vein (arrow head). (D) MIP image from 3D-DSA of the left OA, and shows the shunt point located within the diploic layer (arrow) and draining vein (arrow head).

**Figure 3:**
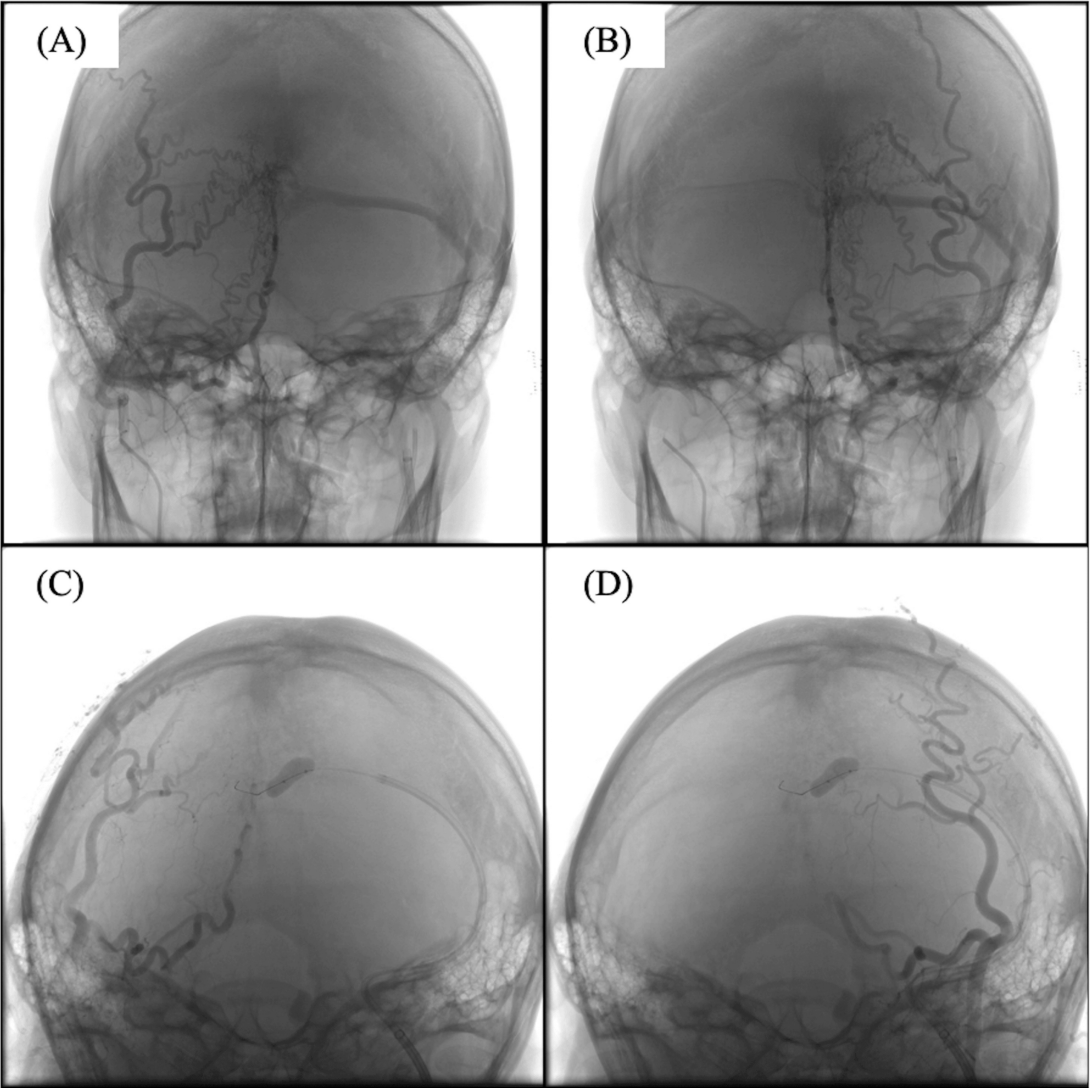
Balloon occlusion test within the transverse sinus confirming a single venous outflow tract. (A) DSA of the right occipital artery (OA) shows that transosseous branches of right OA converged at a single site, forming a shunt that drained into the transverse sinus via a diploic vein. (B) DSA of the left OA shows that transosseous branches of the left OA converged at a single site, forming a shunt that drained into the transverse sinus via a diploic vein. (C,D) Balloon occlusion test performed within the transverse sinus at the orifice of the draining vein. DSA from both OAs showed no residual shunt flow, confirming a single venous outflow tract.

**Figure 4:**
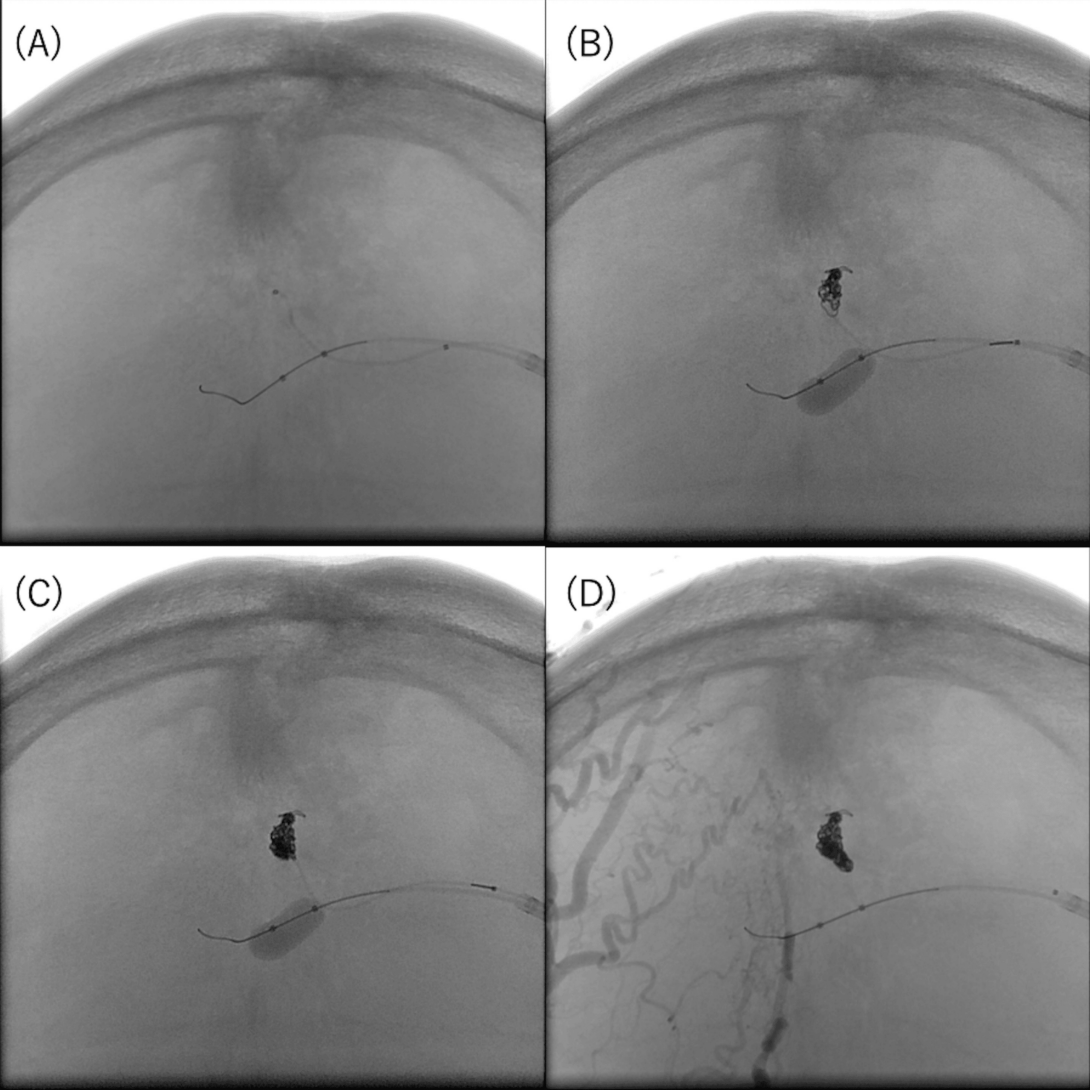
Microcatheter navigation and embolization via the outflow vein. (A) A microcatheter was positioned as close as possible to the shunted pouch. (B,C) Coil embolization was carried out with a microcatheter stabilized by an inflated balloon catheter. (D) Postoperative digital subtraction angiography (DSA) image after completion of coil embolization.

The patient presented with bilateral pulsatile tinnitus as his only complaint. He was alert and exhibited no neurological deficits. There was no significant past medical history, family history, or medication use. Digital subtraction angiography (DSA) revealed arteriovenous shunting via transosseous branches of both occipital arteries (OAs), which converged at a single site within the diploë of the occipital bone and drained into the transverse sinus through a diploic vein (Figure 2). Based on these findings, a diagnosis of intraosseous AVF (diploic AVF) was made. Although venous drainage was antegrade without cortical venous reflux, the patient’s tinnitus was intolerable, and TVE was therefore selected as the treatment (Figures 3-4). TVE was chosen over transarterial embolization (TAE) because both OAs served as arterial feeders, whereas there was a single venous outflow tract that could be directly targeted.

The procedure was performed under general anesthesia. Bilateral femoral arterial access was obtained, and diagnostic catheters were positioned in both OAs for angiography. A guiding sheath was introduced via the right femoral vein and advanced into the left internal jugular vein, where a guiding catheter was placed in the left transverse sinus.

A balloon catheter was positioned inside the transverse sinus and inflated to close the orifice of the draining vein. Angiography confirmed the absence of shunt flow, thereby confirming a single venous outflow tract (Figure 3). 

**Figure 5:**
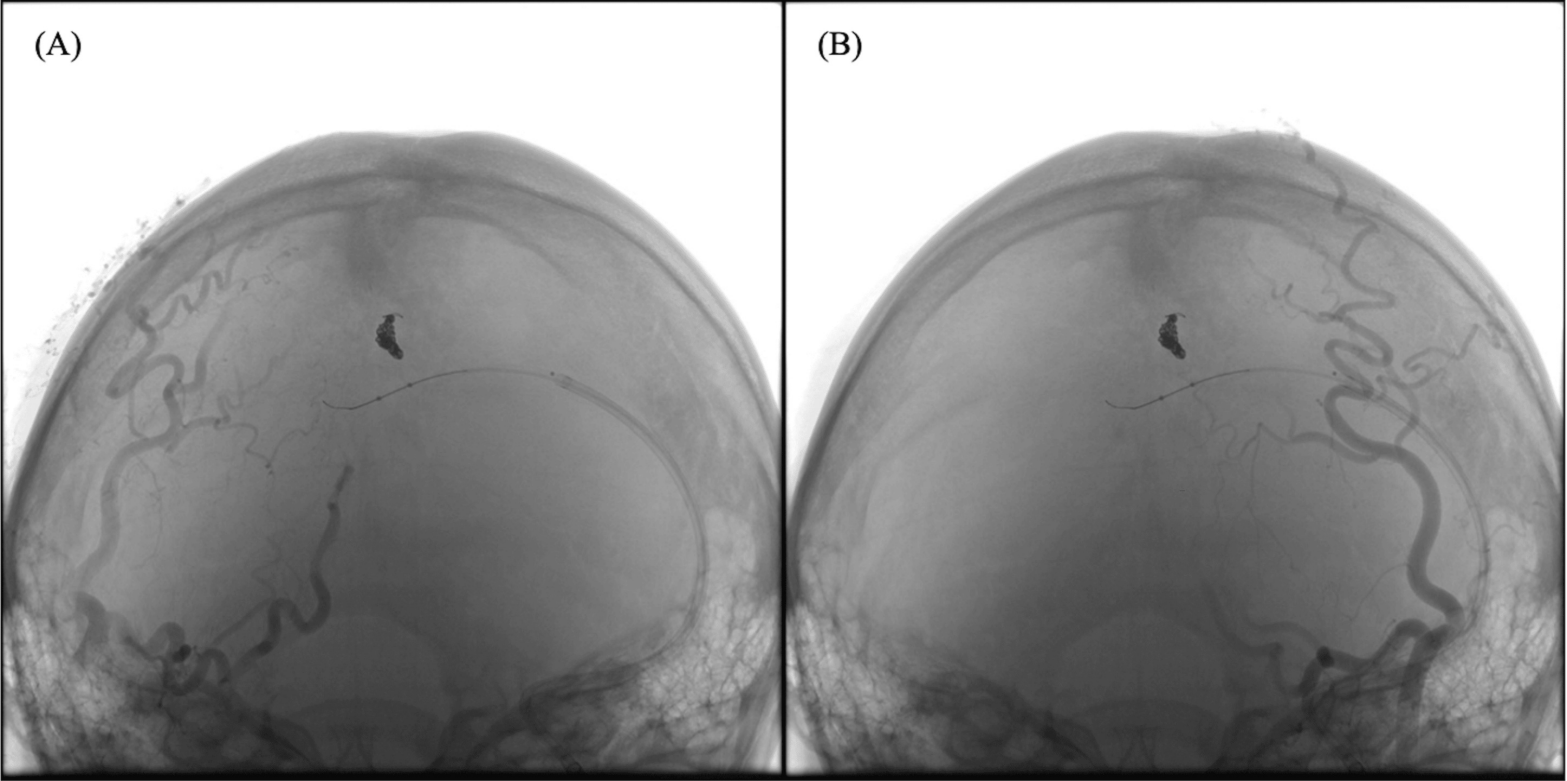
Final digital subtraction angiography (DSA) confirmed complete obliteration of the shunt with preservation of the left transverse sinus and no venous circulation abnormalities. (A) DSA of the right occipital artery (OA) revealed complete resolution of the arteriovenous shunt. (B) DSA of the left OA revealed complete resolution of the arteriovenous shunt.

A microcatheter was navigated into the outflow vein as close as possible to the shunt pouch with the aid of microwires, and embolization was performed (Figure 4). Detachable platinum coils were tightly packed to achieve complete occlusion of the draining vein. Final angiography confirmed complete obliteration of the shunt with preservation of the left transverse sinus and no venous circulation abnormalities (Figure 5).

**Figure 6:**
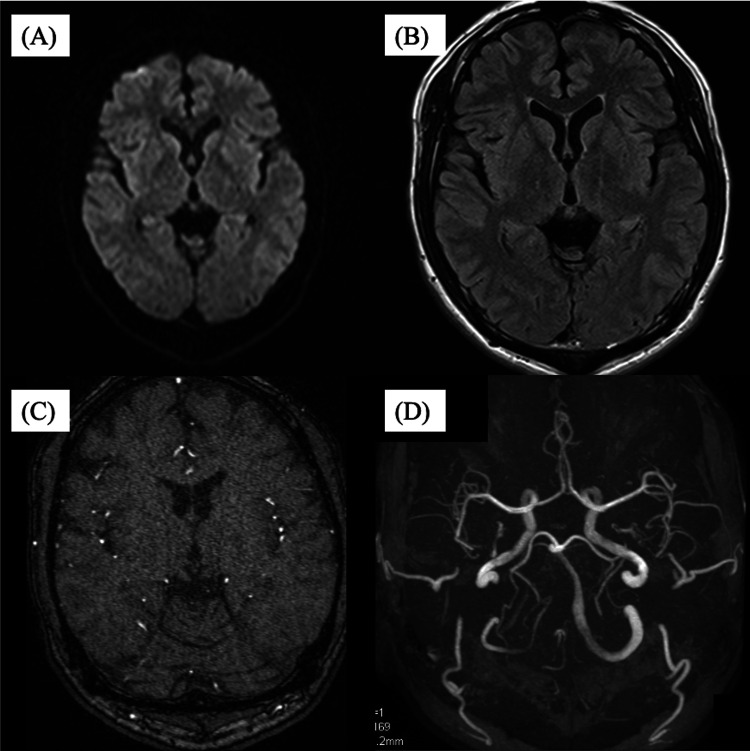
Postoperative MRI and magnetic resonance angiography (MRA). (A) MRI diffusion-weighted imaging (DWI) showed no abnormal findings. (B) MRI fluid-attenuated inversion recovery (FLAIR) showed no abnormal findings. (C) Time-of-flight (TOF)-MRA axial scan demonstrated absence of abnormal flow signal in the left lateral sinus. (D) TOF-MRA 3D reconstruction demonstrated absence of abnormal flow signal in the left lateral sinus.

**Figure 7:**
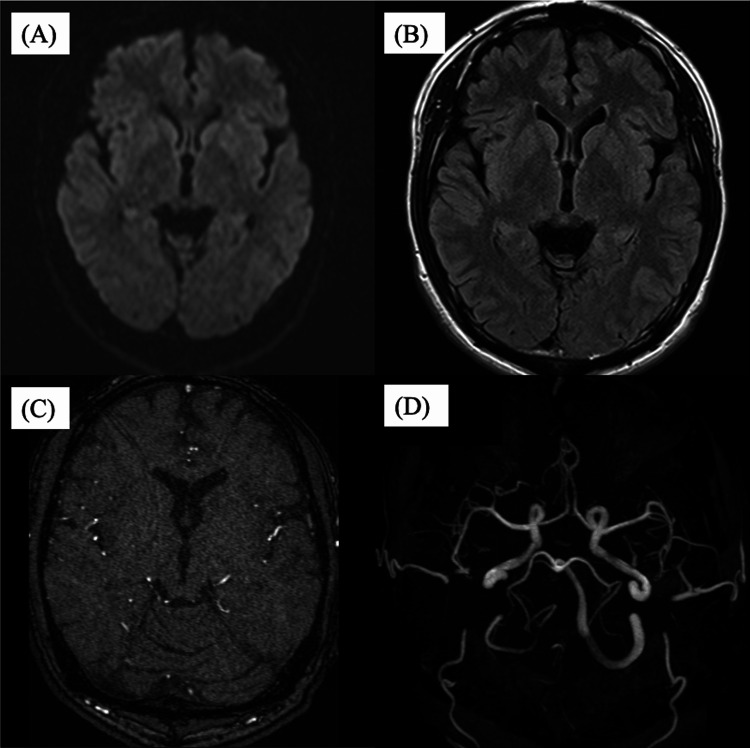
Follow-up MRI and magnetic resonance angiography (MRA) performed six months after treatment. (A) MRI diffusion-weighted imaging (DWI) showed no abnormal findings. (B) MRI fluid-attenuated inversion recovery (FLAIR) showed no abnormal findings. (C) TOF-MRA axial scan demonstrated absence of abnormal flow signal in the left lateral sinus. (D) TOF-MRA 3D reconstruction demonstrated absence of abnormal flow signal in the left lateral sinus.

Postoperatively, the patient’s tinnitus resolved promptly without neurological deficits. MRI showed no new cerebral infarctions, and MRA demonstrated no abnormal signal in the left transverse sinus (Figure 6), which was likewise not visualized on follow-up MRI at six months after the procedure (Figure 7).

## Discussion

Intraosseous AVF is a rare vascular anomaly characterized by an arteriovenous shunt within the diploë. Most previously reported cases have involved the calvaria, particularly the frontal and temporal bones, whereas skull-base lesions are exceedingly rare [1]. When involving the diploic veins, these lesions are termed diploic AVFs. Among them, those affecting the frontal diploic veins are more frequently reported [2-5], whereas cases involving the occipital diploic vein, as seen in our patient, are exceedingly rare [6-8]. This underscores the rarity and clinical relevance of the present case.

Given the limited number of reported cases, the pathogenesis of diploic AVFs remains uncertain. Proposed predisposing factors include idiopathic causes, head trauma [3,9,10], pregnancy [11], and systemic lupus erythematosus (SLE) [10]. In our patient, the absence of relevant medical history supported the diagnosis of an idiopathic diploic AVF.

The clinical presentation of diploic AVFs is variable, including headache, pulsatile tinnitus, and cortical symptoms [2]. In our case, intolerable bilateral pulsatile tinnitus was the primary complaint and prompted further evaluation. This highlights the importance of considering diploic AVF in the differential diagnosis for patients with unexplained pulsatile tinnitus.

Various treatment strategies for diploic AVFs have been reported. Options include TVE with coils and TAE using liquid or particulate agents such as n-butyl cyanoacrylate (n-BCA), Onyx, or polyvinyl alcohol (PVA). Irizato et al. described an occipital intraosseous AVF in an 80-year-old woman, initially observed due to antegrade venous drainage, but later treated with TVE after retrograde venous reflux developed [8]. Fukushima et al. reported successful TAE with n-BCA in a 19-year-old male with a frontal-parietal diploic AVF presenting with headache and nausea [12]. In contrast, Noufal et al. [13] presented a challenging sphenoidal diploic AVF with a large venous aneurysm, where both transarterial and transvenous routes failed. Definitive embolization was ultimately achieved through direct percutaneous cannulation of the sphenoid bone with an 18G needle, followed by embolization with coils and Onyx [13].

Previous reports suggest that the presence of cortical venous reflux or mass effect often dictates the need for intervention. In our case, although cortical reflux was absent, the patient’s quality of life was significantly compromised by severe tinnitus. Identification of the AVF as the clear source of the symptom, combined with the presence of a single, well-defined venous outflow tract, allowed for prompt and effective TVE. This resulted in complete resolution of symptoms and an excellent clinical outcome.

## Conclusions

TVE of an occipital diploic AVF resulted in rapid symptom resolution. This case highlights the importance of considering diploic AVF in the differential diagnosis of pulsatile tinnitus and demonstrates that early targeted intervention, before hemodynamic conditions become complicated, can prevent complications and achieve favorable outcomes.

## References

[1] Jung C, Kwon BJ, Kwon OK, Baik SK, Han MH, Kim JE, Oh CW (2009). Intraosseous cranial dural arteriovenous fistula treated with transvenous embolization. AJNR Am J Neuroradiol.

[2] Tokuyama K, Kiyosue H, Hori Y, Nagatomi H (2020). Diploic arteriovenous fistulas with marked cortical venous reflux. Interv Neuroradiol.

[3] Rivera-Lara L, Gailloud P, Nyquist P (2015). Diploic arteriovenous fistulas—classification and endovascular management. Acta Neurochir (Wien).

[4] Yako R, Masuo O, Kubo K, Nishimura Y, Nakao N (2016). A case of dural arteriovenous fistula draining to the diploic vein presenting with intracerebral hemorrhage. J Neurosurg.

[5] Jo JI, Ryu CW, Ko HC, Shin HS (2022). Direct cannulation of a calvarial diploic vein for embolization of a symptomatic intraosseous arteriovenous fistula: a case report. Taehan Yongsang Uihakhoe Chi.

[6] Sakuma I, Takahashi S, Ishiyama K (2004). Multiple dural arteriovenous fistulas developing after total removal of parasagittal meningioma: a case successfully treated with transvenous embolisation. Clin Radiol Extra.

[7] Hirata K, Kato N, Yamazaki T, Yasuda S, Shiigai M, Matsumaru Y (2021). Arteriovenous fistula of the clival diploic vein associated with arteriovenous fistula of the posterior condylar canal. Interv Neuroradiol.

[8] Irizato N, Asai K, Okubata H, Tateishi A, Taniguchi M, Wakayama A (2024). A case of an intraosseous arteriovenous fistula at the squamous part of the occipital bone with spontaneous occlusion of diploic venous drainage. J Neuroendovasc Ther.

[9] Saba MI, King RB (1973). Extravasation of angiographic contrast material from a torn middle meningeal artery into the diploi. Case report. J Neurosurg.

[10] Guédon A, Eliezer M, Houdart E (2022). Pulsatile tinnitus revealing a diploic arteriovenous fistula: about two cases. Clin Neuroradiol.

[11] Burger IM, Tamargo RJ, Broussard J, Gailloud P (2005). Combined surgical and endovascular treatment of a spontaneous diploic arteriovenous fistula. Case report. J Neurosurg.

[12] Fukushima Y, Matsuda K, Yoshino S, Hirakawa K, Inoue T (2022). A pure acute subdural hematoma presenting with a diploic arteriovenous fistula: case report and literature review. J Neuroendovasc Ther.

[13] Noufal M, Liang C, Chhabra V (2023). Percutaneous transosseous embolization of a diploic vein arteriovenous fistula with intracranial and extracranial shunting. Interv Neuroradiol.

